# WHO working definition of vitality capacity for healthy longevity monitoring

**DOI:** 10.1016/S2666-7568(22)00200-8

**Published:** 2022-11

**Authors:** Ivan Bautmans, Veerle Knoop, Jotheeswaran Amuthavalli Thiyagarajan, Andrea B Maier, John R Beard, Ellen Freiberger, Daniel Belsky, Mylene Aubertin-Leheudre, Christopher Mikton, Matteo Cesari, Yuka Sumi, Theresa Diaz, Anshu Banerjee, WHO Working Group on Vitality Capacity

**Affiliations:** a Gerontology Department and Frailty in Ageing Research Department, Vrije Universiteit Brussel, Brussels, Belgium; b Department of Geriatrics, Universitair Ziekenhuis Brussel, Brussels, Belgium; c Ageing and Health Unit, WHO, Geneva, Switzerland; d Epidemiology, Monitoring, and Evaluation Units, WHO, Geneva, Switzerland; e Department of Maternal, Newborn, Child, and Adolescent Health and Ageing, WHO, Geneva, Switzerland; f Demographic Change and Healthy Aging Unit, Social Determinants of Health, WHO, Geneva, Switzerland; g Department of Human Movement Sciences, @AgeAmsterdam, Amsterdam Movement Sciences, Vrije Universiteit Amsterdam, Amsterdam, Netherlands; h Department of Medicine and Aged Care, @AgeMelbourne, The University of Melbourne, The Royal Melbourne Hospital, Parkville, VIC, Australia; i Yong Loo Lin School of Medicine, Centre for Healthy Longevity, National University of Singapore, Singapore; j National University Health System, Singapore; k Centre of Excellence on Population Ageing Research, University of New South Wales, Sydney, NSW, Australia; l Institute for Biomedicine of Aging, University of Erlangen-Nuremberg, Nuremberg, Germany; m Social Science Research Institute and Department of Medicine, Duke University School of Medicine, Durham, NC, USA; n Centre de Recherche de l’Institut Universitaire de Gériatrie de Montréal, Montreal, QC, Canada; o Faculty of Sciences, Department of Exercise Sciences, Université du Québec à Montréal, QC, Canada

## Abstract

Intrinsic capacity, a crucial concept in healthy ageing, is defined by WHO as “the composite of all the physical and mental capacities that an individual can draw on at any point in time”. Vitality capacity is considered the underlying physiological determinant of intrinsic capacity. To advance the measurement and monitoring of vitality capacity, a working group of WHO staff members and twenty experts representing six WHO regions was convened to discuss and clarify the attributes of vitality capacity and to develop a clear working definition of the concept. Potential biomarkers to measure vitality capacity were identified, and the following consensual working definition was developed: vitality capacity is a physiological state (due to normal or accelerated biological ageing processes) resulting from the interaction between multiple physiological systems, reflected in (the level of) energy and metabolism, neuromuscular function, and immune and stress response functions of the body.

## Introduction

Population ageing is a major demographic transition that presents both opportunities and challenges. From 2000 to 2050, the proportion of the world's population aged 60 years and older will double, whereas the proportion of those aged 80 years and older will almost quadruple.^1^ Although this demographic change is, on the one hand, a triumph of health care and socioeconomic development, on the other hand it poses major challenges for societies because longer lives come with drawbacks. Additionally, important inequalities in life expectancy persist across and within continents and countries.^2^ Healthy longevity depends on policies, health-care systems, and different health determinants.^3^ Therefore, ageing is not identical in each country, which further compounds the heterogeneity in longevity between different regions or countries.

WHO advocates moving away from a disease-focused model of ageing and frailty, and towards a more positive model of healthy ageing that focuses on preserving functional ability and preventing loss of capacity. Healthy ageing is defined by WHO as “the process of developing and maintaining the functional ability that enables well-being in older age”.^1^

Functional ability is then introduced as “all the health-related attributes that enable people to be and to do what they have reason to value”.^1^ Intrinsic capacity is defined by WHO as “the composite of all the physical and mental capacities that an individual can draw on” at any point in time.^1^ Functional ability is determined by intrinsic capacity, but also by environmental factors and by the interactions between them.

Although various domains of intrinsic capacity next to vitality have been proposed (ie, locomotion and psychosocial, sensory, and cognitive^4^, ^5^), no current consensus exists on what such a construct might be composed of, and few metrics have been identified for systematic quantification or monitoring of intrinsic capacity. Most domains of capacity are overt and readily amenable to assessment. Vitality capacity has been suggested as a core domain, representing the underlying physiological component of intrinsic capacity.^6^, ^7^ However, a definition of vitality capacity is still under debate by WHO and others. The term vitality was first used to describe the body functions devoted to metabolising dietary intakes to produce the required amount of energy for the maintenance of optimal homoeostasis.^4^, ^8^ The term was subsequently used to describe the biophysiological status of an individual and the capacity for maintaining homoeostasis in the face of usual daily exposures and of more extreme and unusual or unexpected challenges, such as injury or infection.^9^ Vitality might thus be conceptualised as the amount of intrinsic capacity that can be retained, and could be seen as underlying an individual's vigour, stamina, and resilience to challenges.^10^ However, no consensual, operational definition of vitality capacity has been proposed in the literature so far. The absence of an operational definition results in confusing use of the term in the literature and in a lack of clarity on the measurement tools that might be used to capture or quantify vitality.

If vitality capacity is being considered as the underlying physiological determinant of intrinsic capacity, early detection of deterioration might allow for early interventions to preserve or increase intrinsic capacity. This preservation of intrinsic capacity would enable older people to do “what they have reason to value” and to be active and functional in the society in which they live. Three main characteristics for vitality were proposed by Cesari and colleagues^4^ in a framework for intrinsic capacity: hormonal function, energy metabolism, and cardiorespiratory function. Other attributes such as nutrition, body composition, depression status, respiratory function, muscle endurance, and subjective vitality scales are also proposed in the literature for assessing vitality capacity in older people.^4^, ^11^, ^12^, ^13^, ^14^, ^15^, ^16^, ^17^, ^18^, ^19^ A growing body of evidence from the field of geroscience describes in many ways similar underlying physiological attributes, such acellular senescence, DNA integrity, and intercellular communication.^7^, ^20^

Ageing is accompanied by physical and functional deterioration and by reduction of homoeostatic reserves in multiple physiological systems, leading to a decreased resistance to stressors and to an increased risk of adverse health outcomes—a state called frailty.^18^, ^21^ Frailty is one of the most prominent barriers to healthy ageing and is conceptually defined as “a clinical condition characterized by the individual's increased vulnerability to endogenous and exogenous stressors” by the consensus opinions of experts involved in the Frailty Consensus Conference.^22^ Frailty represents a state of vulnerability to stressors defined by a clinical reduction of reserves, and reduced vitality could be one of the factors leading to frailty (panel 1). Frailty is generally described as a phenotype characterised by a decline of functional and clinical characteristics below a particular threshold (eg, in the Fried Frailty Index^25^), or showing an accumulation of deficits (eg, in the Rockwood Frailty Index^26^). Frailty is integrated in the life course, and frail older adults have a higher risk of decline than robust older adults, leading to an increased risk of adverse outcomes. However, vitality capacity describes continuums that theoretically extend across the life course and that can also evaluate positively. Therefore, Belloni and Cesari^8^ proposed that frailty is a state of deficit accumulation with ageing across the life course. When the available physiological reserves are too low, a person might not be able to cope with any stressor, explaining the trajectory from the pre-frailty state to the frailty state. By evaluating vitality capacity, the functional and physiological reserves of a person can be evaluated. Therefore, vitality capacity and frailty can be seen as related but opposite sides of the spectrum.^8^

Panel 1 **Vitality and frailty: two sides of the same coin?**

Although the debate regarding the conceptual definition of frailty is ongoing, researchers generally agree that frailty is a dynamic, age-related condition characterised by a decline in homoeostatic reserves in multiple physiological systems, which leads to decreased resistance to stressors and increased risk of adverse health outcomes, such as falls, hospitalisation, diminished mobility, increasing disability in activities of daily living and, ultimately, premature death.^23^, ^24^

Despite this heterogeneity, operational frailty scales can be broadly categorised into three main models: frailty instruments measuring physical frailty (based primarily on physical deficits); multidomain frailty instruments (based primarily on a combination of psychological, cognitive, functional, and social deficits); and indexes of accumulated deficits.^24^

Frailty represents a state of susceptibility to stressors defined by a clinical reduction of homoeostatic reserves, and reduced vitality is one of the factors leading to frailty. When vitality represents the reserves of an individual with dynamic trajectories, frailty can be defined as a state of deficit accumulation caused by ageing.^8^ Measuring vitality in the concept of frailty can benefit health-care professionals because it can inform when to take action to reverse the trend towards frailty. Measuring can corroborate clinical decisions by providing objective data that will go beyond the traditional view on frailty; vitality can therefore give an overview of the biological reserves of an individual. Therefore, these two concepts can be seen as complementary; a reduction in vitality can lead to a state of susceptibility to stressors (frailty).

Although several ideas converge on the structure of intrinsic capacity and its subdomains, a clear and consensual working definition of vitality capacity is still lacking. To move towards a positive model of healthy ageing and a focus on preserving functional ability, defining a consensus definition of vitality capacity and its measurements is vital. Therefore, the WHO Ageing and Health Unit, part of the Department of Maternal, Newborn, Child and Adolescent Health and Ageing (Geneva, Switzerland), proposed to bring opinion leaders together in an expert meeting to discuss the operational definition of vitality capacity.

## Expert meeting report

### Purpose

To advance the measurement and monitoring of vitality capacity, the WHO Ageing and Health Unit and the Frailty in Ageing research group at the Vrije Universiteit Brussel (Brussels, Belgium), organised a working group meeting to discuss and clarify the attributes of vitality capacity and to develop a clear conceptual working definition of the term. The meeting was held virtually on Dec 8–9, 2021, gathering WHO staff members and 20 experts (appendix p 2) representing six WHO regions. The aims of the meeting were to review the conceptual framework for vitality capacity in people aged 60 years and older, discuss the attributes of vitality capacity, and develop a working definition of vitality capacity. As a next step, the proposed working definition and the identified attributes for vitality capacity will be used as a reference to conduct a systematic review of the literature on measures that can be recommended for routine monitoring of vitality capacity, which use, for example, data from health information systems and population surveys.

### Methods

During the meeting, presentations on the meeting objectives and background information were given by members of WHO and the Vrije Universiteit Brussel to pave the way for the discussions (appendix p 3). These presentations included the WHO public health framework for healthy ageing, a clinical perspective of vitality capacity, and outcomes of a focused overview of the concepts and attributes of vitality capacity (appendix pp 4–7). This focused overview of the existing concepts and definitions of vitality capacity started from definitions that already exist in the literature, to select existing definitions and proposed attributes of vitality capacity. On the basis of these concepts, a new conceptional definition of vitality capacity could be created, taking into account the concepts that have been published in the literature. A discussion took place on the diagram of vitality developed by Beard and colleagues^7^ (figure). In this diagram, three different levels of vitality capacity are proposed, starting at genetic inheritance, followed by biomolecular systems affected by biological ageing (eg, cellular senescence, glycation, and low-grade inflammation), and higher level physiological systems (including energy homoeostasis, stress response systems, and repair mechanisms). The rate of biological ageing will determine the intervariability in vitality capacity in ageing individuals. In this approach, the three levels are inter-related, which means that the higher level physiological systems are determined by genetic inheritance and biomolecular systems. The experts advised to adapt this model by distinguishing predictors and outcomes of vitality capacity, and to focus on the higher level physiological systems when elaborating a consensus-based working definition. During a virtual brainstorming session, the experts were asked to indicate all potentially relevant attributes of vitality capacity that are related to higher level physiological systems. The large set of attributes were evaluated and grouped under the labels of energy level, fatigue, metabolism, strength, respiratory function, body composition, cardiovascular function, nutrition, and immune system response; several other attributes could not be clustered (panel 2, part A). Several physical tests such as gait speed or sit-to-stand tests were also proposed; however, these tests were not retained because they belong to locomotor capacity, which is another domain of intrinsic capacity. By trying to understand the retrieved attributes and to relate them to higher level physiological systems, three constructs of vitality capacity were proposed: neuromuscular function, energy and metabolism, and immune and stress response functions.

**Figure:**
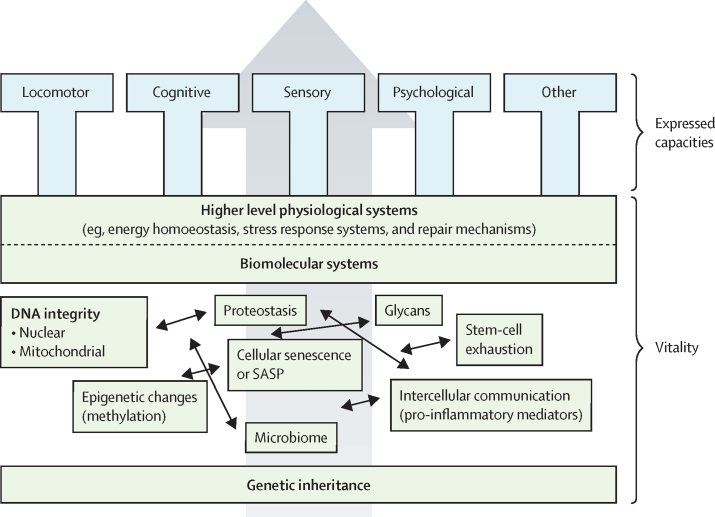
Framework of intrinsic capacity discussed during the expert meeting SASP=senescence-associated secretory phenotype. Reproduced from Beard et al,^7^ by permission of Oxford University Press.

Panel 2 **Stepwise identification of attributes for vitality capacity**

**Part A: Potentially relevant attributes of vitality capacity related to higher level physiological systems**

*Strength and respiratory function*

• Respiratory muscle strength
• Handgrip strength
• Respiratory rate at rest and during exercise

*The level of energy of a person*

• Energy level
• Energy balance
• Energy metabolism
• Energy expenditure

*Fatigue*

• Fatigue symptoms
• Fatiguability
• Muscle endurance
• Daytime fatigue
• Self-perceived fatigue

*Metabolism*

• Insulin sensitivity
• Haemoglobin
• Glycated haemoglobin (HbA_1c_)
• Serum albumin
• Fasting blood glucose
• Hypothalamic–pituitary–adrenal axis hormonal status

*Body composition*

• Anthropometry
• Bodyweight
• BMI
• Waist circumference
• Muscle mass

*Cardiovascular function*

• Heart rate during and after physical activity
• Heart rate variability
• Oxygen saturation
• Orthostatic hypotension
• Orthostatic response after recumbency
• Blood pressure
• Cardiovascular system health
• Maximum oxygen consumption

*Nutrition*

• Nutritional status (Mini Nutritional Assessment)
• Undernutrition
• Malnutrition
• Weight loss
• Loss of appetite

*Others*

• Sympathetic and parasympathetic nervous system function
• Low self-esteem
• Mitochondrial function
• Sedentary behaviour
• Sleep quality
• Methylation clock
• Electrolyte balance

**Part B: Consensus on the top candidate attributes for vitality capacity**

*Energy and metabolism*

• Self-perceived fatigue
• Muscle endurance
• Malnutrition or nutritional status
• Body composition
• Circulating biomarkers of metabolism (eg, HbA_1c_)

*Neuromuscular function*

• Knee extensor strength
• Handgrip strength
• Respiratory muscle strength

*Immune and stress response*

• Circulating biomarkers of inflammation
• Perceived immune status (ie, measured by immune status questionnaire)
• Oxygen saturation
• Autonomic function

Next, experts were divided in three breakout rooms, where they were asked to indicate the top three potential biomarkers for each of these three major constructs of vitality capacity. These biomarkers needed to fulfil the following criteria: feasible to quantify biomarkers or proxy biomarkers; feasible to measure or collect in low-resource settings; useful and informative for monitoring (sufficient sensitivity to change with intervention); distinctiveness (ie, no overlap with other attributes of vitality capacity); acceptable cost and resource demand; sufficient availability and no ethical concerns in collecting and reporting data; and implementable (eg, in dedicated population surveys on ageing or multipurpose surveys; table). These criteria imply that vitality capacity is a dynamic process and, therefore, the selected biomarkers need to be able to detect changes over time. Discussions within these breakout rooms resulted in the identification of several markers: strength (eg, handgrip strength); energy and metabolism markers (eg, albumin and glycated haemoglobin [HbA_1c_] concentrations, malnutrition, waist or calf circumference, fatigue, BMI, inflammation, and haemoglobin concentration); and inflammation and stress response (eg, C-reactive protein concentration, perceived immune status [eg, measured by immune status questionnaire^27^], self-reported immune status, oxygen saturation in combination with strength tests, orthostatic hypotension, and heart rate variability). To reach consensus, we did another virtual exercise during which each expert had to propose their top two candidate markers for neuromuscular function, energy and metabolism function, and immune and stress response function. Discussions following the merging of the breakout rooms resulted in the identification of the biomarkers shortlisted in panel 2, part B.

**Table:** Attributes and criteria applied to potential biomarkers of vitality capacity

	**Feasible to quantify biomarkers or proxy biomarkers**	**Feasible to measure or collect in low-resource settings**	**Useful and informative for monitoring**	**Distinct attribute**	**Acceptable cost and resource demand**	**Sufficient availability and no ethical concerns**	**Implementable**
**Energy and metabolism**							
Self-perceived fatigue	Yes	Yes	Yes	Yes	Yes	Yes	Yes
Muscle endurance	Yes	Yes	Yes	Yes	Yes	Yes	Yes
Malnutrition or nutritional status	Yes	Yes	Yes	Neutral	Yes	Yes	Yes
Body composition	Yes	Neutral	Yes	Yes	Neutral	Neutral	Neutral
Circulating biomarkers of metabolism	Yes	No	Yes	Yes	No	Neutral	No
**Neuromuscular function**							
Knee extensor strength	Yes	Yes	Yes	Yes	Yes	Yes	Yes
Handgrip strength	Yes	Yes	Yes	Yes	Yes	Yes	Yes
Respiratory muscle strength	Yes	Yes	Yes	Yes	Yes	Yes	Yes
**Immune and stress response**							
Circulating biomarkers of inflammation	Neutral	No	Neutral	Yes	No	No	No
Perceived immune status	Yes	Yes	No	Yes	Yes	Yes	Yes
Oxygen saturation	Yes	Neutral	Yes	Yes	Neutral	Neutral	Neutral
Autonomic function	Yes	Neutral	Yes	Yes	Neutral	Neutral	Neutral

Consensus was not reached during the meeting on other possible attributes, such as nutrition, because some of the experts considered it to be a determinant of vitality whereas others considered it an outcome of vitality. Finally, nutrition was included in the vitality framework as the capacity of the body to convert food into nutrients to produce energy, and thus categorised under the energy and metabolism domain.

### Proposed working definition of vitality capacity

To conclude, the following consensual working definition was developed: vitality capacity is a physiological state (due to normal or accelerated biological ageing processes) resulting from the interaction between multiple physiological systems, reflected in (the level of) energy and metabolism, neuromuscular function, and immune and stress response functions of the body.

## Discussion

The current model of health care is designed to anticipate or react to a disease when it manifests clinically, ensuing in medical treatment to eliminate or limit the consequences of the disease. By contrast, the WHO model of intrinsic capacity allows for the identification of people in the community that show either stable or reduced intrinsic capacity, and of those with reduced intrinsic capacity at an early stage, at which point it might be feasible to deliver interventions to prevent further decline and functional losses. Measuring vitality capacity as one of the components of intrinsic capacity can facilitate the monitoring of health trajectories along the life course and across multiple care settings.

Observing the trend in vitality capacity over time (eg, for a whole country) can be informative because these observations can provide information on the factors influencing biological age. Biological ageing reflects the accumulation of damage and compensation for different hallmarks of ageing,^28^ rather than functional limitations.^29^ Cellular and intercellular measures of biological age have been validated against chronological age, and measurements of vitality capacity could thus help to predict an individual's vulnerability to adverse health outcomes on the basis of their level of reserves and functioning rather than their chronological age.^30^, ^31^ Changes over time in vitality capacity might be related to biological ageing, and biomolecular systems are one of the underlying processes that will determine the level of vitality capacity. Interest in the vitality domain of intrinsic capacity is increasing, and debate has been ongoing on whether vitality capacity could be a proper condition for intrinsic capacity, rather than a separate domain. Early detection of deterioration could thus lead to interventions to preserve or increase intrinsic capacity in older people. Several elements in the literature were considered for a new proposed working definition of vitality capacity that could also be linked to healthy longevity. For instance, neuromuscular function, which can be easily quantified by grip strength using a hand-held dynamometer device,^32^ has been suggested as a biomarker of ageing and seems to be a strong predictor for disability (odds ratio [OR] 1·78 [95% CI 1·28–2·48]), falls (1·15 [1·01–1·33]), and mortality (1·79 [1·26–2·55]) in community-dwelling older adults.^33^, ^34^, ^35^, ^36^ In immune and stress response, ageing has been shown to be accompanied by a chronic low-grade inflammatory profile, also known as inflammageing.^37^ Chronic low-grade inflammation is a key element of accelerated biological ageing and of the pathophysiology of frailty and sarcopenia.^19^, ^38^, ^39^ Fatigue is included in the domain of energy and metabolism, and characterises the depletion of physiological reserve capacity^40^ leading to an increased risk of negative health outcomes, such as mortality.^41^ Fatigue has also been shown to be a predictor of several negative health outcomes, such as mortality (OR 2·29 [95% CI 1·67–3·14]), physical disabilities (3·70 [2·80–4·87]), hospitalisation (hazard ratio 1·74 [1·48–2·05]), and physical decline (1.41 (1·28–1·56]) in community-dwelling older adults.^41^ Another study showed that the combination of high self-perceived fatigue and low muscle endurance was associated with a higher risk for frailty.^17^ At the cellular level, energy availability is highly dependent on the function of mitochondria, which are also subject to the ageing process and together with nutritional intake, determine the total energy an individual can use in daily functioning.^42^ Albumin is a marker for nutritional status, and low albumin concentration is a predictor for mortality (with ORs ranging from 7·5 to 12·5).^43^ Although some of the measures of vitality are directly related to biological ageing, others are proxy measures. However, all these markers of vitality capacity reflect biological ageing, which can be accelerated by behavioural and environmental factors.^44^

Vitality capacity is being considered as the underlying physiological determinant of intrinsic capacity and is therefore related to the other domains of intrinsic capacity (figure). The new working definition of vitality capacity and the proposed attributes can consequently influence other intrinsic capacity domains. For example, glycans, such as prolonged concentrations of HbA, are a key factor in neuropathy.^45^ Higher concentrations of HbA_1c_ are also seen in people with diabetes or prediabetes, and this attribute of vitality capacity could thus be related to the sensory domain of intrinsic capacity. The ability to move from one place to another is part of locomotor capacity;^46^ however, sufficient muscular strength is necessary to complete functional tasks (eg, rising from a chair) and is also related to neuromuscular function, which is part of vitality capacity. These examples illustrate how vitality capacity can influence the other domains of intrinsic capacity.

Vitality in the context of frailty can corroborate clinical decisions with objective data that will go beyond the traditional view on frailty. Whereas frailty is based on the accumulation of deficits with ageing, vitality has a more positive approach that advocates moving away from a disease-focused model of ageing. Understanding how vitality capacity affects longitudinal changes in health trajectories is beneficial because it can identify people at risk of developing frailty. Measuring vitality capacity over time can give an indication of the level of a person's reserve capacity; this method is dynamic and it can identify changes in capacity over time. Whereas frailty is a rather static approach of evaluating reserve capacity, vitality capacity can be conceptualised as a continuum measure across the lifespan of older adults. Because vitality capacity can give an overview of the changes in reserve capacity, it is a highly important concept in the context of healthy ageing. Therefore, we assume that these two concepts are complementary; a reduction in vitality can lead to a state of susceptibility to stressors, leading to frailty. Measuring vitality capacity in the context of frailty can benefit health-care professionals by providing them with information on when to take action to reverse the trend towards frailty. Furthermore, measuring vitality at the population level can incentivise clinicians and policy makers to encourage people to remain healthy in old age, by developing policies to promote healthy ageing.

Research has shown that the risks of COVID-19 increases with age.^47^ The COVID-19 pandemic and its associated lockdowns and restrictions might conceivably have accelerated the ageing process substantially,^28^ by contributing to a decline in the vitality capacity of older people, possibly due to a decrease in physical activity during the pandemic.^48^ COVID-19 has therefore highlighted the susceptibility of ageing populations to emerging diseases. SARS-CoV-2 infection is characterised by inflammation, muscle weakness,^49^, ^50^ fatigue, reduced energy levels,^50^, ^51^ and reduced pulmonary diffusion capacity;^50^ all of which are identified as attributes of vitality capacity (panel 3). Therefore, it can be assumed that older adults with reduced vitality are at higher risk of developing COVID-19 than are older adults with higher vitality. This assumption reinforces the importance of monitoring vitality at a population level, allowing appropriate policy decisions to be made during such disease outbreaks and anticipating the effect of associated restrictions on the health of older people.

Panel 3 **Effect of COVID-19 on vitality capacity**

Inflammageing and immunosenescence increase the susceptibility of older people to, and delay recovery from, COVID-19.^52^, ^53^ Fatigue is one of the main symptoms of COVID-19.^51^ However, lockdown restrictions and the lack of physical activity could have also enhanced feelings of fatigue in older individuals. Fatigue is often seen as a marker of age-related accumulation of deficits, representing the underlying vulnerability of an individual's homoeostatic reserves; a sign of biological ageing.^54^ According to Van Kan and colleagues,^55^ when the pandemic is over, many people will show accelerated signs of ageing caused by decline in immune function.

The monitoring of vitality should be implemented at different levels, including by older people themselves (via self-testing or via a proxy of vitality levels), by professional health-care providers in their clinical practice, in scientific research, and by creating anonymised dashboards of vitality levels to support real-time vitality monitoring and policy making. The currently available technology already allows for the detection of clinical signs of frailty and dependency. The aim of measuring and monitoring vitality capacity is to avoid the occurrence and deterioration of symptoms by providing early warning of declining resistance to health stressors. If self-assessment tools (combined with objective tools) become available to measure vitality, older people will be empowered to manage their lifestyle and behaviour and can achieve further agency over their own ageing trajectory. Therefore, simple, non-invasive, and relatively cheap tools that can give a good overview of an individual's vitality capacity should be developed. Health-care professionals need comprehensive longitudinal monitoring of healthy ageing trajectories to estimate risks of adverse health outcomes and to deliver personalised care for older people. Monitoring vitality capacity can shift the curative view on health care towards a more preventive approach, because information regarding vitality capacity is likely to support more tailored interventions and enrich clinical evaluation. Timely identification of older people at risk for declined vitality capacity is essential to provide targeted preventive and health-promoting support to optimise healthy ageing and wellbeing in older people. Measuring vitality at the population level can provide policy makers with information regarding the vitality level and its trajectories in their communities, to investigate which facilities are necessary to enhance healthy ageing. Future research should focus on the optimisation of biomarkers of vitality capacity that can be used in clinical and policy making contexts.

WHO has identified several challenges that must be addressed before vitality capacity can be an integral part of health-care systems designed to promote healthy ageing. These challenges include developing standardised, valid, and objective tools to assess vitality capacity and determining how to monitor this capacity in populations. The current work in vitality capacity can be used as a start; however, more research should be done to identify the measurement tools for evaluating vitality capacity. That should be the focus for the coming years. First, more research should be done to identify the role of vitality capacity across the health span and explore how changes in vitality capacity could improve the functional health span. Second, suitable measures for vitality capacity that are sensitive to responsiveness and applicable at the population level must be identified. Lastly, because vitality can help to target age-related diseases, understanding how vitality can be improved and which long-term interventions can boost vitality capacity to prevent age-related diseases is necessary.

In conclusion, the working group proposed a conceptual working definition that defines vitality capacity as a physiological state (due to normal or accelerated biological ageing processes) resulting from the interaction between multiple physiological systems, reflected in (the level of) energy and metabolism, neuromuscular function, and immune and stress response functions of the body. Defining a conceptual working definition and identifying potential biomarkers for vitality capacity by the experts was the first step. The next step is to perform a systematic review of the potential biomarkers (blood-based, physical, and self-reported biomarkers) of vitality capacity to develop an operational definition of vitality capacity that allows its systematic measurement and monitoring. Further meetings with the working group and projects are planned to expand the operational definition of vitality capacity.

## Declaration of interests

DB reports grants or contracts from the US National Institutes of Health's National Institute on Aging (R21AG054846, R01AG066887, R01AG061378, U01CA164920, R01AG058683, R01AG032282, R01AG057800), the Russel Sage Foundation (BioSS Grant 1810-08987), the Canadian Institute for Advanced Research (CF-0249–CP22-034), Columbia University (Calderone Health Equity Award, Pilot Grant), and Herbert Irving Comprehensive Cancer Center (Pilot Grant); licences from DunedinPACE–TruDiagnostic (no royalties have been received); consulting fees from University of Oslo PROMENTA Center (centre faculty); support for attending meetings or travel from the US National Institutes of Health's National Institute on Aging, Telomere Research Network, Canadian Institute for Advanced Research, University of Oslo PROMENTA Center, and CALERIE Biorepository; participation on a data safety monitoring board or advisory board for Broad River Asset Management; and is a Scientific Advisory Board member at Broad River Asset Management. All other authors declare no competing interests. The authors alone are responsible for the views expressed in this Personal View and does not necessarily represent the views, decisions, or policies of the institutions with which they are affiliated.
