# Supplementary material for: WHO working definition of vitality capacity for healthy longevity monitoring
Source: Lancet Healthy Longev. 2022 Nov;3(11):e789–96. doi: 10.1016/S2666-7568(22)00200-8 (PMC9640935; doi:10.1016/S2666-7568(22)00200-8)
Supplement: Supplementary appendix [file mmc1.pdf]

THE LANCET
Healthy Longevity

Supplementary appendix

This appendix formed part of the original submission and has been peer reviewed. We post it as supplied by the authors.

Supplement to: Bautmans I, Knoop V, Amuthavalli Thiyagarajan J, et al. WHO working definition of vitality capacity for healthy longevity monitoring. *Lancet Healthy Longev* 2022; **3:** e789–96.

Appendix: WHO working definition of vitality capacity for monitoring healthy longevity

Table of Contents

Working group members 2

Meeting agenda: expert meeting day 1 3

Meeting agenda: expert meeting day 2 3

Focused overview of the concepts on definitions and attributes of vitality capacity 4

- Literature search and outcomes 4
- Conceptual framework of vitality capacity 4
- Attributes for measuring vitality found in the literature 5
- Research gaps 5
- References 6

Working group members

Invited experts	
**Name**	**Country**
Andrea Maier	Singapore
Bruno Vellas	France
Cyrus Cooper	United Kingdom
Mylène Aubertin-Leheudre	Canada
Ivan Bautmans	Belgium
Daratt Anantanasuwong	Thailand
Daniel Belsky	United States of America
David Beckwee	Belgium
Ellen Freiberger	Germany
Francesc Gomez-Olive Casas	South Africa
Cesari Matteo	Italy
John Muscedere	Canada
Jeremy David Walston	United States of America
John Beard	Australia
Luis Miguel Gutierrez Robledo	Mexico
Philippe de Souto Barreto	France
Roger Fielding	United States of America
Sabastiana Kalula	South Africa
Stephanie Studenski	Unites States of America
Tahini Altamimi	Saudi Arabia
Veerle Knoop	Belgium
Zee A Han	Republic of Korea
**World Health Organization staff**	
Jotheeswaran Amuthavalli Thiyagarajan	
Theresa Diaz	
Christopher Mikton	
Yuka Sumi	

All members of the vitality capacity working group.

Meeting agenda: expert meeting day 1

Day 1 – 8 December 2021

Time	Agenda	Chair(s) and speakers
3:00 pm to 3:05 pm	Welcome address	Yuka Sumi (WHO)
**Section 1**	**Introduction**	**Chairpersons: Stephanie Studenski (University of Pittsburgh, USA); Ivan Bautmans (Vrije Universiteit Brussel, Belgium)**
3:05 pm to 3:15 pm	Objectives of the meeting and expected outcomes	Jotheeswaran Amuthavalli Thiyagarajan (WHO)
3:15 pm to 3:22 pm	WHO's Public Health Framework for Healthy Ageing	Yuka Sumi (WHO)
3:22 pm to 3:30 pm	Vitality capacity: a clinical perspective	Matteo Cesario (WHO)
3:30 pm to 3:35 pm	Q&A	
**Section 2**	**Conceptual framework and phenotypes of vitality capacity**	**Chairpersons: Stephanie Studenski (University of Pittsburgh, USA); Ivan Bautmans (Vrije Universiteit Brussel, Belgium)**
3:35 pm to 3:45 pm	Conceptual framework and attributes	Veerle Knoop (Vrije Universiteit Brussel, Belgium)
3:45 pm to 4:30 pm	Discussion on the attributes (physiological markers)	Chairpersons
4:30 pm to 4:40 pm	Coffee break	
4:40 pm to 4:50 pm	Review the final list	Chairpersons
4:50 pm to 5:00 pm	Concluding remarks and plans for day 2	Christopher Mikton (WHO); Jotheeswaran Amuthavalli Thiyagarajan (WHO)

Meeting agenda: expert meeting day 2

Day 2 - 9 December 2021

Time	Agenda	Chair(s) and speakers
**Section 2**	**Introduction**	**Chairpersons: Stephanie Studenski (University of Pittsburgh, USA); Ivan Bautmans (Vrije Universiteit Brussel, Belgium)**
3:00 pm to 3:10 pm	Recap of day 1 and outstanding issues	Christopher Mikton (WHO)
3:10 pm to 3:15 pm	Instructions for the breakout session	Jotheeswaran Amuthavalli Thiyagarajan (WHO)
3:15 pm to 3:45 pm	Breakout session	
3:45 pm to 4:00 pm	Coffee break	
4:00 pm to 4:30 pm	Presentations from the groups	Groups leads
4:30 pm to 4:50 pm	Feedback on the conceptual definition	All participants
4:50 pm to 4:55 pm	Next steps	Ivan Bautmans (Vrije Universiteit Brussel, Belgium)
4:55 pm to 5:00 pm	Concluding remarks and the way forward	Theresa Diaz (WHO)

Focused overview of the concepts on definitions and attributes of vitality capacity

Literature search and outcomes

The databases PubMed, Web of Science, Embase, Scopus, PsychInfo, the Cochrane Central Register of Controlled Trials (CENTRAL) and the Cochrane Database of Systematic Reviews (CDSR) are systematically screened for eligible articles. For this preliminary and exploratory literature search only the PubMed database was used. The following key words were used to build a search strategy, firstly we started with including the term "vitality capacity" in the query box of PubMed (Search 29/09/2021) resulting in only 757 articles which there were a few articles including any assessment of "*vitality capacity"* in older people but not on the concept of "*vitality capacity*". This allowed to build a detailed search strategy using different keywords along with MeSH terms to use the PubMed database. The following keywords were used in combination with each other: "Vitality Capacity" AND "Aged" OR "Older adult*" resulting in 174 articles.

Conceptual framework of vitality capacity

Based on the literature resulted from the following keywords: ((Vitality capacity) AND (("Aged"[Mesh]) OR (older adult*))) it was possible to create a framework of vitality definitions of vitality capacity that is used in the literature. This literature search conducted prior to the meeting identified three potential definitions of vitality proposed by WHO collaborators, but none clearly articulated the construct in a way that would support its measurement.

1. The term vitality is used to describe the biophysiological status of an individual and their capacity for maintaining homeostasis in the face of usual daily exposures, as well as more extreme and unusual or unexpected challenges, such as injury or infection *(1)*.
2. Alternatively, vitality could be defined as the body functions devoted to metabolizing dietary intake to produce the required amount of energy to maintain an optimal homeostatic level *(2)*.
3. Vitality might be conceptualized as the amount of intrinsic capacity that can be retained, and it may be seen as underpinning a person's resilience to challenges, as well as their vigour and stamina *(3)*.

Other definitions of vitality were identified such as: vitality is one hand associated liveliness, feeling full or energy and other psychological constructs (4) but also emerges from a physical performance construct of vigor and strength concerning physical domains (5, 6). Ryan and Frederick (4) defined subjective vitality as energy that is perceived to emanate from the self, with an internal locus of causality (extent that one experiences one's energy as "one's own" and as emanating from the self) and is influenced by both psychological and physical factors (7). Giudici, de Souto Barreto (6) created 3 operational definitions of vitality: (1) Vitality representing a mental state of willingness, (2) Vitality corresponding to a physical reservoir (3) and vitality corresponding to global physiological reservoir the fourth quartile for hand grip strength. Vitality can be conceptualized as the amount of IC that can be retained and be seen as underlying a person's resilience to challenges, vigour and stamina (8)
Since vitality has been proposed as the key domain in the construct of intrinsic capacity that drives the other domains it can take place on cellular level as well in higher physiologic systems meaning that when there is an accumulation of deficits this will reach a threshold where overt losses of capacity will be presented.

Attributes for measuring vitality found in the literature

Based on the outcomes of this literature search on the definitions of vitality, the PubMed database was further explores using a more advances search string including the following keywords ((Vitality capacity) AND (("Aged"[Mesh]) OR (older adult*))) AND ((assess* OR screen* OR measur*)) resulting in 134 articles. A first preliminary search led to identification of a number of screening tools for capturing "vitality" including different domains. Energy expenditure and malnutrition vitality, Mental vitality, Physical vitality, Biomarkers for vitality and Self-reported measures of vitality. The objective recording of these parameters mirroring the individual's vitality will predispose the monitoring over time of an essential factor for the phenotypic and functional modifications that the person may experience.

- ***Energy expenditure and malnutrition vitality*** was addressed in the literature through various questions and instruments of witch: resting Metabolic rate(2), weight loss (2, 9), Low body mass index (2, 10), Overweight/obesity(2), question "compared with two years ago, did you lose 5kg or more?(11), question "In the last two years, have you eaten less because of loss of appetite, digestive problems, or difficulties chewing or swallowing?"(11), Abdominal circumference (10, 12), Mini Nutritional assessment (10, 12), mid-upper arm circumference (10), Phase angle derived from bioimpedance measurement (10), loss of appetite (9)
- ***Mental vitality*** was assessed in the literature through questions from the Geriatric Depression Scale (13) in two articles(7, 14).
- ***Physical vitality*** consisted of handgrip strength (7, 10, 12, 14-18), Peak flow test (8, 10, 15, 16), cycle ergometer (14), Grip Work (19, 20), fatigue resistance test(20) and Capacity to Perceived Vitality parameter (20).
- ***Biomarkers for vitality*** in the literature including: Dehydro-epiandrosteron (DHEA), Insulin-like growth factor (IGF-1), full blood assay and hemoglobin (10, 14-16).
- ***Subjective assessment tools for vitality*** including the Subjective Vitality Scale (SVS) (21, 22), MMSE (23) and self-reported fatigue(19), LASA-FI (24)

Research gaps

As shown in the previous sections its clearly that there is a lack of a clear conceptual and operational definition of the vitality domain; no consensual operational definition of vitality has been proposed in literature so far. The lack of a definition makes that in the literature many related constructs could be found, and that is unclear which measurement tools can capture vitality.

Experts taking part to this meeting are therefore expected to reach consensus on the best applicable instruments that can be used on national level to measure the concept of "*vitality capacity*", which, as shown, seems to have not been extensively studied so far as such. Therefore, the aims of the meeting will be to (i) review the conceptual framework for vitality capacity in older people, (ii) discuss the attributes of vitality capacity and (iii) develop a working definition of vitality capacity.

References

1. WHO. DECADE OF HEALTHY AGEING BASELINE REPORT2020.
2. Cesari M, Araujo de Carvalho I, Amuthavalli Thiyagarajan J, Cooper C, Martin FC, Reginster JY, et al. Evidence for the Domains Supporting the Construct of Intrinsic Capacity. J Gerontol A Biol Sci Med Sci. 2018;73(12):1653-60.
3. Cesari M, Sadana R, Sumi Y, Amuthavalli Thiyagarajan J, Banerjee A. What Is Intrinsic Capacity and Why Should Nutrition Be Included in the Vitality Domain? J Gerontol A Biol Sci Med Sci. 2022;77(1):91-3.
4. Ryan RM, Frederick C. On energy, personality, and health: subjective vitality as a dynamic reflection of well-being. J Pers. 1997;65(3):529-65.
5. Sternang O, Reynolds CA, Finkel D, Ernsth-Bravell M, Pedersen NL, Dahl Aslan AK. Factors associated with grip strength decline in older adults. Age Ageing. 2015;44(2):269-74.
6. Giudici KV, de Souto Barreto P, Soriano G, Rolland Y, Vellas B. Defining Vitality: Associations of Three Operational Definitions of Vitality with Disability in Instrumental Activities of Daily Living and Frailty among Elderly Over a 3-Year Follow-Up (MAPT Study). J Nutr Health Aging. 2019;23(4):386-92.
7. Masciocchi E, Maltais M, El Haddad K, Virecoulon Giudici K, Rolland Y, Vellas B, et al. Defining Vitality Using Physical and Mental Well-Being Measures in Nursing Homes: A Prospective Study. J Nutr Health Aging. 2020;24(1):37-42.
8. Arokiasamy P, Selvamani Y, Jotheeswaran AT, Sadana R. Socioeconomic differences in handgrip strength and its association with measures of intrinsic capacity among older adults in six middle-income countries. Sci Rep. 2021;11(1):19494.
9. Ramirez-Velez R, Correa-Bautista JE, Garcia-Hermoso A, Cano CA, Izquierdo M. Reference values for handgrip strength and their association with intrinsic capacity domains among older adults. J Cachexia Sarcopenia Muscle. 2019;10(2):278-86.
10. George PP, Lun P, Ong SP, Lim WS. A Rapid Review of the Measurement of Intrinsic Capacity in Older Adults. J Nutr Health Aging. 2021;25(6):774-82.
11. Gutiérrez-Robledo LM, García-Chanes RE, Pérez-Zepeda MU. Screening intrinsic capacity and its epidemiological characterization: a secondary analysis of the Mexican Health and Aging Study. Rev Panam Salud Publica. 2021;45:e121.
12. Charles A, Buckinx F, Locquet M, Reginster JY, Petermans J, Gruslin B, et al. Prediction of Adverse Outcomes in Nursing Home Residents According to Intrinsic Capacity Proposed by the World Health Organization. J Gerontol A Biol Sci Med Sci. 2020;75(8):1594-9.
13. Yesavage JA, Brink TL, Rose TL, Lum O, Huang V, Adey M, et al. Development and validation of a geriatric depression screening scale: A preliminary report. Journal of Psychiatric Research. 1982;17(1):37-49.
14. Gonzalez-Bautista E, Andrieu S, Gutiérrez-Robledo LM, García-Chanes RE, de Souto Barreto P. In the quest of a Standard Index of Intrinsic Capacity. A Critical Literature Review. J Nutr Health Aging. 2020;24(9):959-65.
15. Beard JR, Jotheeswaran AT, Cesari M, Araujo de Carvalho I. The structure and predictive value of intrinsic capacity in a longitudinal study of ageing. BMJ Open. 2019;9(11):e026119.
16. Beard JR, Si Y, Liu Z, Chenoweth L, Hanewald K. Intrinsic Capacity: Validation of a New WHO Concept for Healthy Ageing in a Longitudinal Chinese Study. J Gerontol A Biol Sci Med Sci. 2021.
17. Huang CH, Umegaki H, Makino T, Uemura K, Hayashi T, Kitada T, et al. Effect of Various Exercises on Intrinsic Capacity in Older Adults With Subjective Cognitive Concerns. J Am Med Dir Assoc. 2020.
18. Giudici KV, de Souto Barreto P, Beard J, Cantet C, Araujo de Carvalho I, Rolland Y, et al. Effect of long-term omega-3 supplementation and a lifestyle multidomain intervention on intrinsic capacity among community-dwelling older adults: Secondary analysis of a randomized, placebo-controlled trial (MAPT study). Maturitas. 2020;141:39-45.
19. Knoop V, Cloots B, Costenoble A, Debain A, Azzopardi RV, Vermeiren S, et al. Fatigue and the prediction of negative health outcomes: a systematic review with meta-analysis. Ageing Res Rev. 2021:101261.
20. Knoop V, Costenoble A, Debain A, Azzopardi RV, Vermeiren S, Laere SV, et al. The interrelationship between grip work, self-perceived fatigue and pre-frailty in community-dwelling octogenarians. Exp Gerontol. 2021:111440.
21. Rouse PC, Veldhuijzen Van Zanten JJ, Ntoumanis N, Metsios GS, Yu CA, Kitas GD, et al. Measuring the positive psychological well-being of people with rheumatoid arthritis: a cross-sectional validation of the subjective vitality scale. Arthritis Res Ther. 2015;17:312.
22. Park S, Park K, Hancox J, Castaneda-Gameros D, Koo KC. Physical Activity and Subjective Vitality in Older Adults From Community-and Assisted-Living Settings. Asian Nurs Res (Korean Soc Nurs Sci). 2019;13(5):313-7.
23. Minicuci N, Marzari C, Maggi S, Noale M, Senesi A, Crepaldi G. Predictors of transitions in vitality: the italian longitudinal study on aging. J Gerontol A Biol Sci Med Sci. 2005;60(5):566-73.

24. de Breij S, van Hout HPJ, de Bruin SR, Schuster NA, Deeg DJH, Huisman M, et al. Predictors of Frailty and Vitality in Older Adults Aged 75 years and Over: Results from the Longitudinal Aging Study Amsterdam. Gerontology. 2021;67(1):69-77.
